# Occurrence and associated factors of aflatoxin M1 in raw cow milk in South Gondar Zone, North West Ethiopia, 2020

**DOI:** 10.1002/fsn3.2589

**Published:** 2021-09-22

**Authors:** Fitalew Tadele Admasu, Addisu Melak, Biruk Demissie, Chalachew Yenew, Mezgebu Legesse Habtie, Tigist Tefera Bekele, Teka Obsa Feyesa, Ermias Sisay Chanie, Markeshaw Tiruneh G/Medhin, Tabarak Malik, Tadesse Asmamaw Dejenie

**Affiliations:** ^1^ Department Medical Biochemistry College of Health Sciences Debre Tabor University Debre Tabor Ethiopia; ^2^ Department of Social and Public Health College of Health Sciences Debre Tabor University Debre Tabor Ethiopia; ^3^ Department of Biochemistry, School of medicine College of Health and Medical Sciences Haramaya University Harar Ethiopia; ^4^ Department of Pediatrics and Child Health Nursing Debre Tabor University Debre Tabor Ethiopia; ^5^ Department of Biotechnology The institution of Biotechnology University of Gondar Gondar Ethiopia; ^6^ Department of Biochemistry School of Medicine College of Medicine and Health Sciences University of Gondar Gondar Ethiopia

**Keywords:** aflatoxins, dairy farmer, ELISA, ethiopia, raw milk

## Abstract

Aflatoxin M1 is the most significant toxin of milk and milk products. It is immunosuppressive, mutagenic, and carcinogenic compounds to humans. Therefore, this study was aimed to evaluate the concentration of aflatoxin M1 and its determinants in raw cow milk sample intended for human consumption in South Gondar Zone, Ethiopia. A cross‐sectional study was conducted on a total of 100 dairy farmers from January to February 2020. Around 50 ml, 100 raw milk samples were collected for aflatoxin M1 analysis. A simple random sampling technique was applied to get the households. Binary and multivariate logistic regressions were used to see the association between predictor and outcome variables. From the 100 dairy farmers who had participated, 38% had heard about aflatoxin in the milk sample. Aflatoxin M1 was detected in the 99(99%) raw milk samples, of these 41 (41%) exceeded the limit of the European Union. The logistic regression analysis result showed that residence, awareness about the level of aflatoxin in the milk sample, management mold‐contaminated animal feed, animal feed storage facility, and grazing systems were significantly associated with the high level of aflatoxin in the milk sample. Almost all milk samples analyzed were positive for aflatoxin M1, and 41% of samples were above the limit set by European Union. Many easily manageable and preventable factors were associated with higher levels of aflatoxin M1 in the milk sample than the European Union limit, which suggests continuous monitoring of milk and milk products is necessary.

## Graphical abstract

This work is targeted at assessing Aflatoxin M1 in raw milk and its associated factors.This manuscript has not been published and is not under consideration for publication elsewhere.

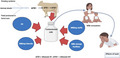

## 1 INTRODUCTION

Milk is an important and relatively cheap source of diet, which contains diverse macro‐ and micronutrients to sustain life (Kagera et al., 2018). The dairy sector in developing countries makes up to 40% of the agricultural gross domestic product (GDP) and 4% of the national GDP (Serraino et al., 2019). Most of the total milk production is consumed on‐farm or marketed informally (direct sale of raw milk from the farm to consumers, wholesale distributors, retailers, and cooperative societies) and only a small percentage is processed and marketed formally. Milk is mainly consumed as tea or gruel, as raw or after boiling it (Kagera et al., 2018; Serraino et al., 2019). Aflatoxin M1 (AFM1) is the most significant toxin in milk and dairy products, and its heat‐stable nature makes it difficult to destroy during processing (World Health Organization and Joint FAO/WHO Expert Committee on Food Additives, 2020; Milićević et al., 2019). Annually around 25% of food and food products are af fected by mycotoxins. Aflatoxins are highly toxic secondary metabolites (World Health Organization and Joint FAO/WHO Expert Committee on Food Additives, 2020; Ghajarbeygi et al., 2016; Gonçalves et al., 2019). Aflatoxin B1, B2, G1, and G2 are the main classes, and aflatoxin M1 and M2 are hydroxylated metabolic end‐products of B1 and B2, respectively, which can present in urine and milk of animals fed on aflatoxin‐contaminated feeds (Lalah et al., 2019).

The most common disease‐causing aflatoxin is AFB1 which has carcinogenic and genotoxic effects potentially. Aflatoxin contamination can occur in any animal‐fed processing (from the field, storage, and transportation). Staple foods (wheat and maize), groundnuts, cassava, oilseeds (cotton, sunflower), fruits, wines, legumes, milk, and milk products may be contaminated by aflatoxins (Gonçalves et al., 2017; Wu & Turna, 2019). AFM1 is a hydroxylated metabolite of AFB1 that may be found in milk and milk products of livestock that have ingested contaminated feed, and there is a linear relationship between the amount of AFM1 in milk and AFB1 in feed consumed by animals (Kamkar et al., 2011). An animal that consumes aflatoxin in feed converts it to AFM1 at a rate of about 2.5% (Gizachew et al., 2016; Wu & Turna, 2019). The International Agency for Research on Cancer (IARC) has classified AFB1 and AFM1 as class I carcinogens (Gizachew et al., 2016). Feeding of readymade concentrate and leftover household cereals, longer feed storage duration, and feed storage quality can contribute to the presence of AFM1 in farm milk (Gizachew et al., 2016; Serraino et al., 2019).

Several international studies have found higher levels of AFM1 in milk. Infants and children are the most susceptible group due to the high level of milk consumption, and their biochemical detoxification mechanisms are not fully operative (Costamagna et al., 2019). Long time exposure of children to aflatoxins can result in growth retardation, immune suppression, growth impairment especially stunting, leading the child to increased susceptibility to infections and cognitive impairments (Unnevehr et al., 2013).

Milk is one of the most important exposure factors to AFM1. Many countries have implemented regulatory legal limits for mycotoxins in food, especially for AFM1 in raw milk and milk products (Ghajarbeygi et al., 2016; Gonçalves et al., 2017). The limit varies from not detectable to 15 µg/L. 0.05 µg/L and 0.5 µg/L are the two most common regulatory limits.

The European Commission establishes the maximum permitted level for AFM1 as 0.025 µg/kg for infant formulae and 0.05 µg/kg for raw milk (Perugini et al., 2009). Ethiopia uses the regulatory limits set by the European Commission (0.05 µg/kg for raw milk). The US Food and Drug Administration has set the AFM1 in dairy products at 0.5 µg/L level (Ruangwises et al., 2013).

Ethiopia has the highest cattle populations in Africa, estimated at 60 million heads and around 90% of milk comes which are important sources of dietary nutrients for infants, children, convalescents, and old people (Tafere & Hassen, 2012). In Ethiopia, investigations on the occurrence of aflatoxin in the milk sample and dairy products are scarce. Therefore, this study was aimed to determine the concentration of AFM1 and identifying factors associated with raw fresh cow milk in South Gondar Zone, Ethiopia.

## 2 METHODS AND MATERIALS

### 2.1 Study design and period

A laboratory‐based cross‐sectional study was conducted among dairy farmers living in South Gondar Zone from January to February 2020. A simple random sampling technique was applied to get dairy farmers; accordingly, 100 private diary farmers were recruited.

### 2.2 Study participants

A total of 100 private dairy farmers living in and around Debre Tabor (urban and rural), south Gondar Zone, were participated as a study population using 93% proportion (study conducted in Addis Ababa) (Gonçalves et al., 2017), 95% confidence interval, and 5% margin of error.

### 2.3 Milk sample collection and sample preparation

Up to 50 ml of untreated raw fresh cow milk samples was collected from all of the 100 private diary farmers by sterile screw‐capped plastic tubes. After collection, all milk samples were placed in an insulated cold box and carried to the laboratory and kept in the refrigerator at −20°C until laboratory analysis. All milk samples were analyzed for AFM1 before the expiration date of the samples.

A pretested structured questionnaire developed by reviewing many international and national studies in the Ethiopian context was used to capture data on animal feeding and feed storage practices and farmers’ awareness of AFM1(Chaisri et al., 2017; Gizachew et al., 2015; Kiama et al., 2016).

### 2.4 Aflatoxin M1 determination

The quantitative measurement of AFM1 was done using high sensitivity commercial enzyme‐linked immunosorbent assay (ELISA; Biotech Instruments, Inc.,) that detects AFM1 in the concentration range between 0.05 µg/L and 0.1 µg/L (Bio scientific, 2008) at Bahir Dar University Institute of Technology, Food Chemistry Laboratory. Samples that exceeded the highest standard (0.1 µg/L) were diluted using skim milk (aflatoxin free) provided in the kit and retested in duplicates. The assay was done by following the protocol provided by the manufacturer. The milk sample was centrifuged at 2000 rpm for 5 min to allow separation of the upper fatty layer, supernatant was separated, and the excess was used for analysis. The upper fatty layer was removed, and the lower skimmed milk layer was used in the assay. Finally, the optical density (OD) of each was read with a microplate reader at 450 nm using an air blank. The AFM1 level in each was calculated using a logarithmic standard curve and the average of the duplicates used as the final results.

The accuracy and precision of the analysis were evaluated by the % recovery (measured concentration/fortified concentration) *100 and % coefficient of variation (standard deviation/mean) *100, respectively, and both were found in an acceptable range.

### 2.5 Data processing and statistical analysis

The data were entered into Epi‐Data version 3.1 and then exported to SPSS (version 21.0) for analysis. Aflatoxin M1 levels in milk were categorized into legal and high based on the laboratory results through comparing internationally accepted limits set by European Commission (0.05 µg/L). Percentage, frequency, mean, and standard deviation were calculated. Binary logistic regression analysis was used to assess statistical associations between the predictor and the outcome variables. Those variables with *p*‐value <0.25 in the bivariable logistic regression analysis were included in multivariable logistic regression analysis. *p*‐value less than or equal to 0.05 was considered statistically significant, and an odds ratio with 95% confidence intervals was used to examine associations between predictors and outcome variables.

### 2.6 Data quality assurance

To validate the data, the experiment was done in triplicate. Sample collection, handling, storage, and extraction were made based on scientific protocols, and all milk sample analysis was done based on scientific standard laboratory procedures (proper handwashing, wearing latex gloves, and laboratory coats). Proper sterilization and disinfection techniques of instruments were made based on international standard procedures.

## 3 RESULT

### 3.1 Sociodemographic and household characteristics

The number of cattle owned by the households ranged between 1 and 12 (4 ± 3) per household. On average, 57.5% of the owned animals were lactating and being milked at the time of sample collection. Most farmers (94%) milked their cows twice a day. The daily milk production was 15.8 ± 13 L/day/households and 0.25–27 liters/cow/day.

### 3.2 Feeding practice (animal feed types, sources, storage)

The majority of farmers are practicing mixed grazing 61(61%) followed by 27(27%) zero‐grazing practices. In most of the household (78%), dairy animals were intensively managed and were supplemented with commercial concentrates. The majority of dairy farmers (94% and 93%) sourced their animal feed from hay and cut–carry–pasture, respectively (Table 1). Most dairy farmers (88%) stored animal feeds and routinely monitored their conditions such as temperature, ventilation, moisture, mold growth, dryness, and pests. While 68% of dairy farmers would throw away feeds if they noticed mold growth, 32% of them, feed to their animals after they expose to air. Among the total dairy farmers, 73% hay, 75% cut–carry–pasture, and 80% concentrates stored on a raised surface, respectively (Table 1).

**TABLE 1:** Dairy farmers’ animal feed type, source, storage condition, and grazing system, in South Gondar Zone, Northwest Ethiopia, 2020

Type	Source		Storage		
Feed type	Dairy farmer (Households)%	% On‐farm formulation	% Purchased	%Stored on the floor	%Stored on a raised surface
Hay	98	11	88	22	77
Cut–carry–pasture	97	52	47	20	80
Concentrates	98	7	92	27	72
Silage	56	100	0	66	33
Grazing system	Frequency	Percent (%)			
Zero grazing	27%	27%			
Open grazing	12%	12%			
Mixed grazing	61%	61%			

### 3.3 Knowledge and practices related to the level of aflatoxin in the milk sample

From 100 visited households, 75% of them thought that milk safety can be determined by senses, mainly by sight and taste, and 25% thought that senses alone cannot determine the safety of milk (Table 2). Most farmers (90%) did not know the availability of milk safety tests like lactometer tests that could show the safety of milk. Most dairy farmers (69%) stored milk in clear plastic buckets, 11% of them stored in cold places including cold areas within the house or immersing containers with milk in cold water, and only 15% of dairy farmers stored their milk in the refrigerator. Around 98% of the respondents reported that milk could be contaminated by, contaminated animal feeds, storage temperature, milk contaminant, milk storage utensils, and animal illness and can result in milk spoilage. In the present study, 20% of the respondents discarded their milk when it spoiled, 15% gave the milk to their pets, 48% of them made butter and discarded the milk, and 17% heated and used it. Of the total respondents (100), only 38% had heard about the level of aflatoxin in the milk sample, of these, only 14% described correctly and the rest 24% were not able to correctly describe, while 62% of the respondents had no information on aflatoxins. Around 32% of the respondents believed that the presence of aflatoxin in milk and other products pose danger to human health (Table 2).

**TABLE 2:** Dairy farmers' knowledge and practices about aflatoxins in South Gondar Zone, Northwest Ethiopia, 2020 (*n* = 100)

Variables	Frequency	
How do you know about milk safety	Senses	75
	Need tests	25
Do you know milk safety tests	Yes	10
	no	90
How do you store milk	Refrigerator	15
	Plastic buckets	69
	Aluminum cans	5
	Cold place	11
Dose milk can be contaminated	Yes	98
	No	2
Experience milk spoilage	Yes	78
	No	22
What do you do to spoiled milk	Discard	20
	Give to animals	15
	Make butter	48
	Heat and use	17
Heard about aflatoxin in the milk sample	Yes	38
	No	62
Knowledge about the causes of aflatoxin in the milk sample (*n* = 38)	No	8
	Contaminated animal feed	8
	Storing animal feed in Moist place	20
	Other	2
Is aflatoxin have a health problem(*n* = 38)	No	6
	Gastrointestinal problem	20
	Liver problem	6
	Poisoning	5
	Death	1

### 3.4 Aflatoxin M1 contamination in raw milk

A total of 100 milk samples (20 from urban and 80 from rural areas) were collected and analyzed for AFM1. AFM1 was detected in 99 analyzed milk samples, with values ranged from 0.031 µg/L to 5.16 µg/L (mean = 0.47 µg/L), and one sample from the rural residence was below the limit of detection (LOD) of aflatoxin analyzer (0.02 µg/L). Among 20 milk samples collected from periurban (Debre Tabor), 13 (65%) were above 0.05µg/L. From all milk samples, 52 from rural residence and 7 from Debre Tabor, AFM1 was less or equal to 0.05 µg/L. Furthermore, 41 (41%) (28 from a rural area and 13 from Debre Tabor) AFM1 exceeded 0.05 µg/L, which is the legal limit allowed by the European Union (Table 3). Delete the red one trade as it is redundant or already mentioned above.

**TABLE 3:** AFM1 level of milk sample among dairy farmers found in South Gondar Zone, Northwest Ethiopia, 2020 (*n* = 100)

AFM1 (µg/l)	(Rural area, *n* = 80)	(Debre Tabor, *n* = 20)	Total(*n* = 100)
Mean	0.41	0.68	0.47
Standard deviation	0.71	0.85	0.73
Median	0.097	0.33	0.98
Minimum	<LOD	0.045	<LOD
Maximum	4.89	5.16	5.16
≥0.05 µg/L	28	13	41
≤0.05 µg/L	52	7	59

### 3.5 Factors associated with aflatoxin in the milk sample contamination

Bivariate logistic regression analysis showed that residence, awareness about the level of aflatoxin in the milk sample, management of animal feed with mold growth, feed storage facility, the quantity of milk, grazing system, and concentrate supplementation were significantly associated with milk AFM1 level ≥0.05 µg/L.

However, in multivariate logistic regression analysis only rural residence, poor awareness about the level of aflatoxin in the milk sample, feeding animal feed contaminated by mold, unavailability of animal feed storage facility, and mixed grazing systems were significantly associated with AFM1 level above the level set by the EU.

Milk samples from periurban areas had 6.4 (AOR = 6.4:95% CI: 3.4, 9.2) times more likelihood of being contaminated (≥0.05 µg/L) by AFM1 compared with rural residences. The analysis result also indicated that AFM1 level was fivefold higher in dairy farmers who had no awareness about the level of aflatoxin in the milk sample (AOR = 5.5:95%: CI: 1.7, 8.1) than those who had awareness. Dairy farmers who had to expose feed contaminated by mold to air and give back to their animals were 5.8 times (AOR = 5.8:95% CI: 3.3, 8.2) more likely to have a higher level of AFM1 than those who had not given. Furthermore, farmers who had no animal feed storage facility were 5.2 times (AOR = 5.2:95% CI: 1.9, 8.9) more likely to have high AFM1 levels in milk than those who had a storage facility. Dairy farmers who had practiced a mixed grazing system have higher (AOR = 7.5, 95%CI: 0.6, 10.6) levels of AFM1 compared to zero and open grazing systems (Table 4).

**TABLE 4:** Bivariate and multivariate logistic regression analyses of factors associated with AFM1 level ≥0.05 µg/L among dairy farmers in South Gondar Zone, Northwest Ethiopia, 2020 (*n* = 100)

Factor	the level of aflatoxin in the milk sample		95% CI		*p*‐value
	≤0.05 µg/L	≥0.05 µg/L	COR (95%CI)	AOR (95%CI)	
Residence					
Urban(town)	7	13	1.8(0.9, 3.4)	6.4(3.4, 9.2)	0.036*
Rural	52	28	1.0	0.1	
Awareness about the level of aflatoxin in the milk sample					
Yes	16	22	1.0	1.0	
No	43	19	4.7(1.7, 12.8)	5.5(1.7, 8.1)	0.001*
What do you do to animal feeds with mold growth					
Throw away	45	23	1.0	1.0	
Air and give back	14	18	13.5(1.5,23.7)	5.8(3.3, 8.2)	0.011*
Having an animal feed storage facility					
Yes	54	34	1.0	1.0	
No	5	7	2.4(1.2, 4.5)	5.2(1.9, 8.9)	0.031*
Quantity of milk					
Large	36	42	7.8(0.4, 33.1)	7.8(0.6, 13.76)	0.098
small	47	195	1.0	1.0	
Grazing system					
Zero grazing	15	12	1.8(0.2, 16.6)	8.6(0.2, 52.9)	0.779
Open grazing	5	7	1.0	1.0	
Mixed grazing	38	23	16.8(11.5,23.8)	7.5 (0.6, 10.6)	0.03*
Concentrate supplementation					
Yes	49	29	2.9 (2.4, 6.3)	8.2 (2.4, 15.6)	0.517
No	10	12	1.0	1.0	

Note

^∗^The association is significant at a *p*‐value ≤of 0.05.

## 4 DISCUSSIONS

This study was conducted to evaluate the presence and level of AFM1 and associated factors among private diary farmers of the south Gondar zone.

Generally, there was low knowledge of aflatoxins among dairy farmers that only 38% had heard of the level of aflatoxin in the milk sample and 90% of them did not know the availability of quick milk safety tests. This result was low when compared to studies conducted in Wolaita Ethiopia (Kibret et al., 2019), Nairobi (Kubokaa et al., 2019), and Kenya (Kiama et al., 2016). Among the 100 milk samples, 99(99%) were contaminated with AFM1. The AFM1 contamination level obtained in this study was comparable with many studies (Hussain et al., 2008; Gizachew et al., 2016; Costamagna et al., 2019). However, higher than the other studies conducted in Egypt (Ismaiel et al., 2020) and two studies from Turkey (Yilmaz et al., 2019). The mean value of AFM1 of this study was comparable with the study conducted in Addis Ababa but higher than the result reported from Peru (Puga et al., 2020) and Argentina (Costamagna et al., 2019) and lower than the study found in Egypt (Temamogullari et al., 2014). This variation in milk AFM1 level could be due to milk originating from different agroecological conditions, applying different feeding practices, different climate conditions, different cattle management systems, feed storage conditions, and different levels of awareness on milk AFM1. From the total milk samples, 41 (41%) were exceeded the limit allowed by European Union which was higher than studies conducted in Egypt (Tahoun et al., 2017) and Kenya (Kagera et al., 2018) and lower than a study conducted in Iran (Kamkar et al., 2011).

Several factors may impact the occurrence and level of AFM1 in milk and milk derivatives. In this study being periurban residence had 6.4 times more likely to have a higher level of AFM1 than the rural residence. The finding was supported by a study conducted in Kenya (Makau et al., 2016) and reasoned that lengthy storage of on‐farm formulated concentrates because of shortage of animal feed and lack of grazing fields in urban areas.

Urban dairy farmers may practice concentrate feeding because of the inaccessibility of other feeding mechanisms, storing animal feed in a restricted, and closed room, keep cows within a confined grazing area that may favor, and facilitate mold growth and contamination of feed by AFM1(Soler et al., 2010). Furthermore, urban and periurban countries may lack diversification of food items which may balance the susceptible and unsusceptible animal feeds as against monolithic susceptible animal feed (Anthony et al., 2016).

Dairy farmers who did not know the level of aflatoxin in the milk sample have a fivefold higher level of AFM1 in milk samples when compared to those who have. The study on the farmers’ knowledge of fungal toxins showed that 62% of dairy farmers had never heard of the level of aflatoxin in the milk sample. Low knowledge and unclear concept about aflatoxins are common in many developing countries, which may allow high aflatoxin exposure through contaminated feeds leading to human disease (Ayo et al., 2018). Dairy farmers with awareness about the level of aflatoxin in the milk sample may have information on the source and will protect feed and cows from AFB1 exposure and ultimately safeguard milk from contamination.

The study also showed that dairy farmers who fed mold‐contaminated animal feed to their animals after airing and exposure to sunlight had five times more prone to have higher milk AFM1 than those who discarded it. This finding is supported by another study (Chaisri et al., 2017). Cows usually excrete AFM1 in their milk within twelve hours of consumption of mold‐contaminated animal feeds (Makau et al., 2016). This might be due to physical methods such as airing and sunlight exposure might not completely remove mold from the feed and may increase the level of AFM1 in milk.

In this study, a mixed grazing system was also an important determinant of high milk AFM1. Dairy farmers who practice mixed grazing systems showed 7.5 times higher risk of high AFM1 concentration in milk than those who used zero and open grazing systems. Here, the reason probably during mixed grazing system concentrate feeding is more applicable and dairy farmers who feed concentrate for their cattle increase the probability of having a high level of AFM1 in the milk sample. A Kenyan study relieved that feeding concentrates are more likely to have a high level of AFM1 than others (Anyango et al., 2018).

Households that had no animal feed storage facility were 5.2 times more likely to have higher AFM1 in milk when compared to households that have a storage facility. Feed which are not kept in raised and ventilated platforms, storing in indoor plastic bags, and longtime (more than 6 months) storage condition may create a conducive environment for the accumulation of molds and aflatoxins (Anthony et al., 2016; Gizachew et al., 2015). Having no storage or improper storage conditions such as high humidity and high temperature, fungal growth is favored, and there is an increased risk of mycotoxin in milk (Chaisri et al., 2017).

## 5 CONCLUSION

Dairy farmers’ awareness about the level of aflatoxin in the milk sample was poor, 99% of milk samples were contaminated by AFM1, and 41% were above the recommended level of EU. Many animals’ feed storage and related factors were associated with higher milk AFM1 levels. Therefore, educating dairy farmers on the prevention mechanisms and lethal effects of mycotoxins during all stages of animal feed preparation and feeding should be given. Moreover, animal feeds and dairy products should be screened regularly.

## CONFLICT OF INTERESTS

The authors declare that they have no competing interests.

## AUTHORS CONTRIBUTION

**Fitalew Tadele Admasu:** Conceptualization (equal); Data curation (equal); Formal analysis (equal); Methodology (equal); Writing‐original draft (equal). **Addisu Melak:** Investigation (equal); Supervision (equal); Visualization (equal). **Biruk Demissie Melse:** Formal analysis (equal); Supervision (equal); Validation (equal); Visualization (equal). **Chalachew Yenew Denku:** Supervision (equal); Writing‐review & editing (equal). **Mezgebu Legesse Habtie:** Supervision (equal); Writing‐review & editing (equal). **Tigist Tefera Bekele:** Formal analysis (equal); Investigation (equal). **Teka Obsa Feyesa:** Supervision (equal); Validation (equal). **Ermias Sisay Chanie:** Investigation (equal); Supervision (equal). **Markeshaw Tiruneh G/Medhin:** Investigation (equal); Methodology (equal); Validation (equal). **Tabarak Malik:** Investigation (equal); Resources (equal); Supervision (equal); Visualization (equal). **Tadesse Asmamaw Dejenie:** Formal analysis (equal); Methodology (equal); Writing‐review & editing (equal).

## ETHICAL APPROVAL

Ethical approval was obtained from the Institutional Review Board, the research committee of Debre Tabor University, College of Health Sciences. A sampling of cow milk was carried out with the full consent of the head of the household.

## ARRIVE GUIDELINE

This study was carried out in compliance with the ARRIVE guideline recommendations for animal study.

## CONSENT TO PUBLISH

Not applicable.

## Data Availability

All data generated and analyzed during this study are available from the corresponding author at a reasonable request.
